# Deferoxamine Modulates Corneal Endothelial Cell Biological Properties Associated with Increased VEGF Expression

**DOI:** 10.3390/medicina62050808

**Published:** 2026-04-23

**Authors:** Barbara Sophie Brunner, Paul Emesz, Nikolaus Luft, Siegfried Georg Priglinger, Andreas Ohlmann, Stefan Kassumeh

**Affiliations:** Department of Ophthalmology, University Hospital, LMU Munich, Mathildenstrasse 8, 80336 Munich, Germany

**Keywords:** corneal endothelial cells, corneal endothelial regeneration, deferoxamine, vascular endothelial growth factor

## Abstract

*Background and Objectives:* The objective of this study is to evaluate whether deferoxamine modulates cell biological properties, such as proliferation and wound closure of porcine corneal endothelial cells (CECs) in vitro, and whether the treatment of CECs with deferoxamine results in an enhanced expression of vascular endothelial growth factor (VEGF). *Materials and Methods:* Corneal endothelial cells were extracted from porcine globes within 24 h postmortem. Immunohistochemistry for the endothelial Na^+^/K^+^-ATPase was performed to confirm the cells’ endothelial origin. To assess CEC viability and proliferation, a water-soluble tetrazolium salt (WST-1) and 5-bromo-2′-deoxyuridine (BrdU) assay were performed. Corneal endothelial wound closure was evaluated using a wound closure assay. VEGF mRNA expression was evaluated using real-time polymerase chain reaction (rt-PCR). *Results:* The extracted corneal endothelial cells showed a typical hexagonal morphology with Na^+^/K^+^-ATPase staining of the cell membrane. The treatment with 200 µM deferoxamine significantly increased CEC viability to 121 ± 24% compared to the control group (*p* = 0.0024). Corneal endothelial cell proliferation did not show any significant changes under the treatment with deferoxamine (*p* > 0.05). Both 100 µM and 200 µM deferoxamine led to a significantly smaller remaining wound area of 82.4 ± 6.7% and 78.7 ± 6.2% (*p* < 0.0001) in comparison to the control group after 24 h of treatment in the wound closure assay. Treatment with 200 µM deferoxamine significantly induced VEGF mRNA expression to 1.67- ± 0.57-fold from 1.00- ± 0.03-fold in the control group (*p* = 0.0006). *Conclusions:* Deferoxamine effectively enhances corneal endothelial cell viability and wound healing associated with an overexpression of VEGF. Thus, deferoxamine is a potent modulator of cell biological properties of corneal endothelial cells and maintains their integrity in vitro.

## 1. Introduction

Proliferation and migration of corneal endothelial cells (CECs) are essential for recovery of the corneal endothelium and recreation of visual acuity after ocular trauma, intraocular surgery, or corneal endothelial dysfunction, for example in Fuchs’ endothelial corneal dystrophy (FECD). In FECD, a step-by-step decrease in the corneal endothelial cell density, a thickening of the Descemet membrane and the deposition of extracellular matrix, in the form of so-called guttae, occurs. Eventually, due to apoptosis, a corneal endothelial cell loss of function leads to a disbalance of the corneal hydration status and subsequent corneal swelling [1,2].

Currently, the standard of care is the replacement of the affected corneal endothelium and its underlying Descemet membrane with human donor tissue (Descemet membrane endothelial keratoplasty; DMEK) [3]. Unfortunately, when removing the Descemet membrane, the natural scaffold for corneal endothelial cells to migrate onto the wound area is eliminated. Therefore, an autonomous recovery of the corneal endothelium by migrating towards the bare corneal stroma without further support or a lamellar corneal graft is unlikely [4,5].

Recent experimental and clinical investigations indicate that rho-kinase inhibitors, such as ripasudil, may sufficiently induce corneal endothelial cell recovery following excimer laser debridement of corneal endothelium ex vivo [6] or manual descemetorhexis without introduction of a corneal graft in FECD patients in vivo [7]. However, this requires the corneal endothelial cells to migrate onto the blank affected corneal stroma area [8,9]. In addition, CECs naturally show a very limited potential to proliferate and thus are not able to compensate for damage to the corneal endothelium [10]. To eventually establish a treatment option without a corneal graft, a pharmacological substance that not only enhances CEC migration but also proliferation is of importance.

Hypoxia is a common physiologic and pathologic occurrence in humans [11]. In addition to fetal development [12,13], adaption to altitude [14] and the regulation of the epithelial barrier function of the gastric mucosa [15], hypoxia has a key role in different disease entities. It is documented to be prominent in processes such as inflammation, wound healing, atherosclerosis, stroke and the development of solid tumors [16]. The avascular cornea and the corneal endothelium are especially affected by hypoxia-induced stress by reduction of cell count, the development of guttae and corneal edema [17].

Under normoxic conditions, hypoxia-inducible factor is constantly degraded by specific HIF hydroxylases [18,19]. In contrast, hypoxic conditions lead to a stabilization of HIF-1α and increase nuclear transcription via different pathways [16,20]. Eventually, adaption to hypoxia through promotion of angiogenesis, glycolysis, erythropoiesis, and adaptive cellular processes (for example progenitor cell recruitment) is achieved [21]. However, a destabilization of HIF-1α impairs local fibroblast function, neovascularization and distal progenitor cell recruitment that can cause deficiencies in aged and diabetic wound healing [17,22,23,24].

Targeting hypoxia-induced signaling pathways during wound healing has many important clinical implications for tissue repair [25]. Counteracting the detrimental effects of excessive or deficient hypoxia-induced signaling by modulating HIF-1α expression may improve future management of poorly healing wounds, such as the corneal endothelium following descemetorhexis without introducing a corneal graft [26].

Deferoxamine, an FDA-approved iron-chelating substance, has been in clinical use for hemochromatosis treatment for decades [27]. Previously, studies indicated that deferoxamine may have potential applications in the rising field of tissue regeneration due to its unique properties comprising a downregulation of inflammation and promotion of vascularization, and thus, enhancement of wound healing in vivo [28]. A recent study found that a topical treatment with a deferoxamine solution improved wound healing in diabetic and aged mice [29]. On a molecular biological basis, deferoxamine significantly inhibits HIF-1α degradation and consecutively increases HIF-1α levels. Thus, it leads to a higher expression of vascular endothelial growth factor (VEGF) [30]. In addition to its positive effects on angiogenesis via HIF-1α, deferoxamine seems to also mediate wound healing via a reduction of reactive oxygen species (ROS), which are present in high concentrations in tissue during inflammation [28].

Therefore, deferoxamine may represent a potential candidate for further investigation as an agent for perioperative treatment in FECD patients or as a preventive therapy for corneal endothelial decompensation after intraocular surgery and ocular trauma. To this end, we evaluated whether deferoxamine enhances proliferation and corneal endothelial wound healing of porcine corneal endothelial cells in vitro and further, whether the treatment of CECs with deferoxamine leads to an overexpression of VEGF, a downstream target that can be associated with hypoxia-induced signaling pathways.

## 2. Materials and Methods

### 2.1. Corneal Endothelial Cell Extraction and Cell Culture

Corneal endothelial cells were extracted from 30 porcine globes, which were obtained from a local butchery following approval by the authority for waste management of animal carcasses (ID: DE 09 162 0066-21) and processed within 3 to 5 h postmortem. During transport, a sufficient cooling with cool packs was maintained. To avoid epithelial contamination, a complete abrasion of the corneal epithelium was done with a hockey knife (Katena Products, Inc., Parsippany, NJ, USA). Subsequently, sclerocorneal discs were cut out and placed with the epithelium face-down into a 6-well cell culture plate (NUNC, Langenselbold, Germany). The preformed ‘cup’ was filled with approximately 250 µL 2.5% trypsin (Merck Millipore, Burlington, MA, USA). Following 5 min of incubation at 37 °C, CECs were gently detached using a 1000 µL pipette and transferred to a clean well. CECs were kept in cell culture medium (Dulbecco’s MEM, DMEM; Merck Millipore, Burlington, MA, USA) supplemented with 10% fetal bovine serum (Merck Millipore, Burlington, MA, USA), 50 IU penicillin/mL and 50 µg streptomycin/mL at 37 °C and 5% CO_2_. Corneal endothelial cells (CECs) were used exclusively at early passages (≤passage 3) and for a maximum of 10 days, as later passages showed phenotypic drift, characterized by loss of endothelial cell morphology and a switch towards a fibroblast-like morphology.

### 2.2. Immunohistochemistry

For immunohistochemistry, corneal endothelial cells were cultured on circular cover glasses with a diameter of 12 mm (Karl Hecht GmbH & Co. KG, Sondheim, Germany) until subtotal cell coverage. Following fixation with 4% paraformaldehyde for 10 min, CECs were washed and hydrated in 0.1 M phosphate buffer for another 10 min at room temperature. Blocking was conducted by applying 3% bovine serum albumin (BSA) and 0.1% Triton X-100 in 0.1 M phosphate buffer for 60 min. Consequently, for staining against Na^+^/K^+^-ATPase, incubation with an Anti-Na^+^/K^+^-ATPase antibody conjugated with Alexa Fluor 488 (1:50; Merck Millipore, Burlington, MA, USA) in 0.3% BSA and 0.01% Triton X-100 in 0.1 M phosphate buffer for 24 h at 4 °C was performed. Following another washing step, nuclear staining was performed using 1:50 4′,6-diamidino-2-phenylendole (DAPI; Vector Laboratories Inc., Burlingame, CA, USA). Mounting was performed using ProLong Glass Antifade Mountant (Thermo Fisher Scientific Inc., Waltham, MA, USA). Images were acquired on an Axiovision fluorescence microscope (Axio Observer 7; Carl Zeiss, Jena, Germany).

### 2.3. Corneal Endothelial Cell Viability and Proliferation

For assessment of porcine corneal endothelial cell viability, a water-soluble tetrazolium salt (WST-1) cell proliferation assay (Hoffmann-La Roche AG, Basel, Switzerland) was performed. Approximately 10,000 cells were seeded onto each well of a 96-well cell culture plate (NUNC, Langenselbold, Germany) and incubated with supplemented DMEM. Upon confluence, cells were kept under supplement-free conditions for 24 h before treatment with different concentrations of deferoxamine (DFO; deferoxamine mesylate, Selleckchem Chemical LLC, Houston, TX, USA) dissolved in dimethyl sulfoxide (DMSO; Carl Roth AG, Karlsruhe, Germany) for another 72 h. Control groups were supplemented with DMSO only. Eventually, the cells were incubated with the WST-1 solution for 60 min. Substrate turnover was measured with an ELISA reader at 450 nm (Spectramax 190; Molecular Devices, Sunnyvale, CA, USA) and a reference at 690 nm.

Proliferation of CECs was determined using a 5-bromo-2′-deoxyuridine (BrdU) ELISA according to the manufacturers’ recommendations (Merck KGaA, Darmstadt, Germany). Therefore, 7000 corneal endothelial cells were transferred to a 96-well cell culture plate and incubated under standard conditions for 24 h. Following another incubation period of 24 h under supplement-free conditions, CECs were treated with deferoxamine or DMSO in an equivalent concentration. Cells were then incubated with BrdU labeling solution for 2 h and subsequently fixed with 4% paraformaldehyde and anti-BrdU antibodies in accordance with the manufacturer’s recommendations. BrdU incorporation into the DNA of CECs was quantified by absorbance measurement at 450 nm.

### 2.4. Corneal Endothelial Wound Closure

To analyze porcine corneal endothelial cell wound closure, cells were cultured in DMEM supplemented with fetal calf serum until confluence of the cells of a 6-well cell culture plate. Consequently, cells were kept under serum-free conditions for 24 h before 5 linear scratches per well were performed using a 200 µL pipette tip (Eppendorf AG, Hamburg, Germany). Prospective incubation for 24 h under the treatment with deferoxamine or DMSO as a control followed. Reference images of 4–5 different scratch sections were acquired at the beginning of the incubation period using the phase contrast mode on the Axio Observer 7 (Carl Zeiss, Jena, Germany) with a follow-up after 24 h. The size of the wound area was analyzed using a semi-automated analysis tool in ImageJ 1.53a (National Institute of Health, Bethesda, MD, USA).

### 2.5. RNA Isolation, cDNA Synthesis and Real-Time Polymerase Chain Reaction Analysis

For mRNA expression analysis, the total RNA of porcine corneal endothelial cells treated with deferoxamine for 12 h was isolated with peqGOLD TriFast (VWR, Radnor, PA, USA) according to the manufacturers’ instructions. The RNA concentration and the OD260/OD280 ratio were measured with the Biophotometer^®^ (Eppendorf AG, Hamburg, Germany). Only an optical density ratio between 1.6 and 2.0 was considered suitable for single-strand DNA synthesis, which was performed by using the iScript cDNA Synthesis Kit (Bio-Rad Laboratories Inc., Hercules, CA, USA) in accordance with the manufacturers’ recommendations. Real-time PCR was conducted on a CFX real-time PCR detection system (Bio-Rad Laboratories Inc.). Polymerase chain reaction was performed in a volume of 15 µL using the 2× SYBR Green Master Mix (Bio-Rad Laboratories Inc.). The thermocycler profile was 40 cycles at 95 °C for 10 s for denaturation and 40 s of annealing and extension at 60 °C. All primer pairs of the candidate genes span exon–intron boundaries. For expression analysis of VEGF-mRNA, the primer pair 5′-TCTACCTCCACCATGCCAAG-3′ and 5′-TGGGGTTTCTGGTCTCCTTC-3′ was used and for GNB2L mRNA, the primer pair 5′-GATTGCTACCACTCCCCAGT-3′ and 5′-TGGTCTCATCTCTGGTCAGC-3′ was used (all by Thermo Fisher Scientific Inc., Waltham, MA, USA). GNB2L served as the housekeeper for relative quantification. Results were analyzed using the CFX Manager Software Version 3.1 (Bio-Rad Laboratories Inc.). Samples with an OD260/280 ratio ≥ 1.6 were only included if the downstream quality control parameters (amplification efficiency, melting curve analysis, and housekeeping gene stability) were within the reference range of the manufacturer’s guidance.

### 2.6. Data Handling and Statistical Analysis

For all assays, between three and nine independent experiments were performed. Each independent experiment was conducted with porcine CEC from a different globe at different time points. For each independent experiment, up to 5 replicates were generated. Replicates were derived from the same cell line (same globe) but seeded onto different cell culture wells.

All results are expressed as mean ± standard deviation. A one-way analysis of variance (ANOVA) was performed to compare the mean variables of more than 2 groups. A Tukey post hoc test followed to correct for multiple comparisons. To depict significant changes over time (wound healing assays) a two-way ANOVA with Geisser–Greenhouse correction was conducted. Normality of the data distribution was assessed for all treatment groups using the D’Agostino–Pearson omnibus test. Across all treatment groups, results indicated no significant deviation from a Gaussian distribution (*p* > 0.05), supporting the assumption of normality for subsequent parametric analyses. *p*-values less than 0.05 were considered as statistically significant. Graphs were plotted with Prism 8 (GraphPad Software, Version 10.6.1, San Diego, CA, USA).

## 3. Results

### 3.1. Corneal Endothelial Cell Morphology and Na^+^/K^+^-ATPase Expression

**Figure 1:**
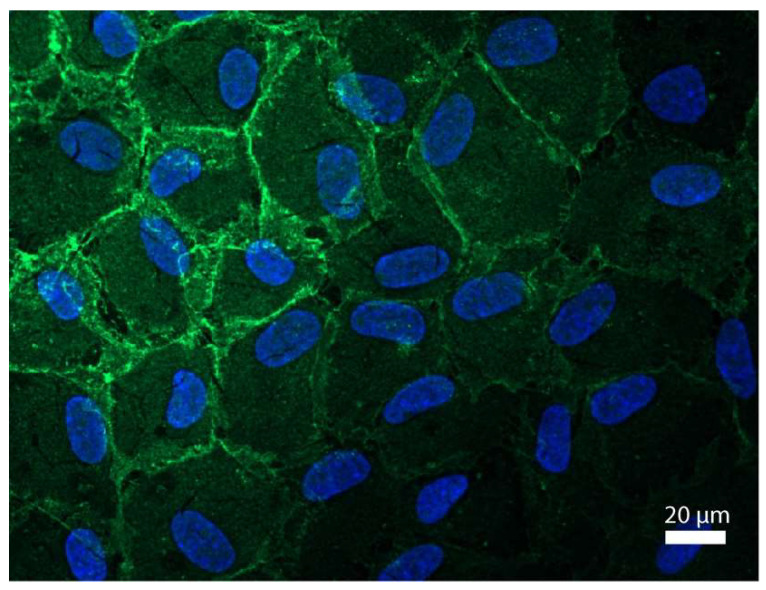
Hexagonal corneal endothelial cells with Na^+^/K^+^-ATPase staining of the cell membrane. Representative image of corneal endothelial cells stained against Na^+^/K^+^-ATPase (green) and nuclear staining with DAPI (blue). Magnification: 40×; scale bar: 20 µm.

Figure 1 shows a representative image of corneal endothelial cells stained against Na^+^/K^+^-ATPase and nuclear staining with DAPI (blue; Figure 1). A distinct hexagonal structure in a dense cell formation with a higher fluorescence intensity (green; Figure 1) of the cell membrane of corneal endothelial cells could be observed. No staining for Na^+^/K^+^-ATPase of the cell nuclei occurred.

### 3.2. Deferoxamine Enhances Corneal Endothelial Cell Viability

**Figure 2:**
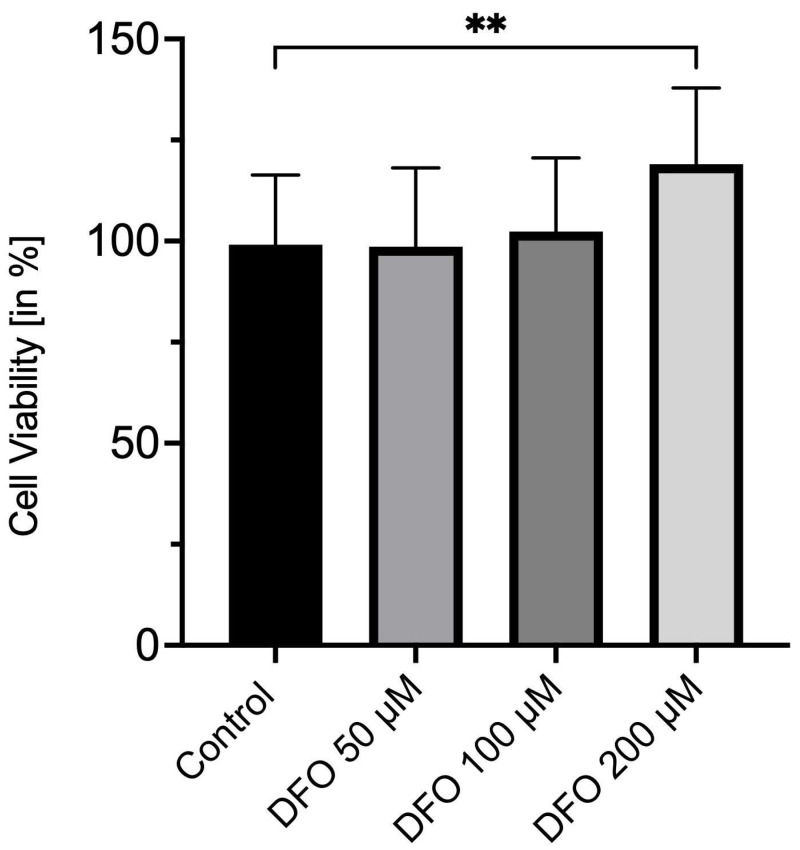
Deferoxamine enhances corneal endothelial cell viability. WST-1 cell viability assay of corneal endothelial cells treated with deferoxamine [50 µM, 100 µM and 200 µM] or DMSO only as a control group for 72 h. ** *p* < 0.01. Co: control; DFO: deferoxamine. *n* = four independent experiments with five replicates per experiment.

After 72 h of incubation, corneal endothelial cells treated with 200 µM deferoxamine showed a significant increase in cell viability to 121 ± 24% (Figure 2), when compared to the control group treated with DMSO (100 ± 17%; *p* = 0.0024). Figure 2 further depicts a significantly higher cell viability of CECs under the impact of 200 µM deferoxamine instead of 50 µM (99 ± 19%; *p* = 0.0020) or 100 µM (101 ± 18%; *p* = 0.0041). No significant changes in cell viability were observed between 50 µM and 100 µM in comparison to the control group (for both groups *p* = 0.9828, Figure 2).

### 3.3. No Proliferative Effects of Deferoxamine on Corneal Endothelial Cells

**Figure 3:**
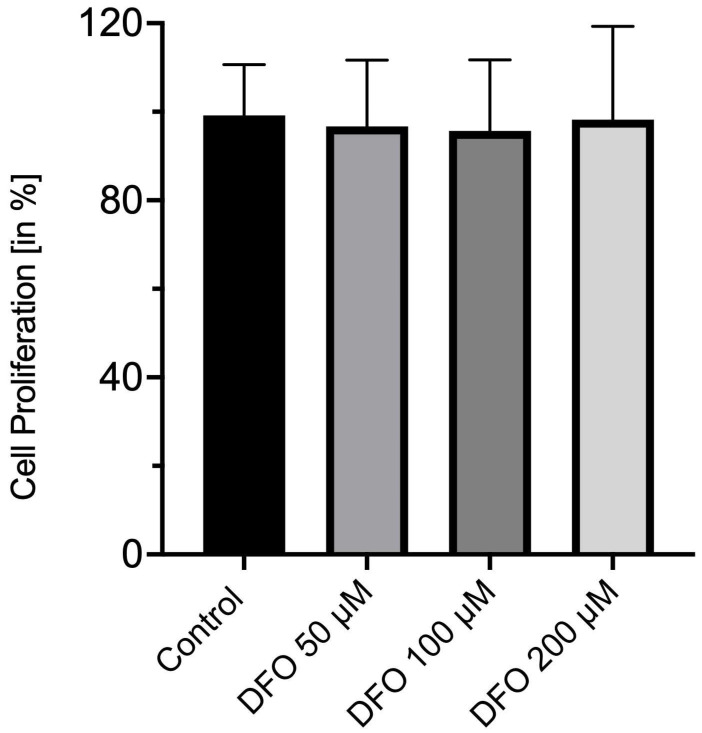
Deferoxamine has no effect on corneal endothelial cell proliferation. BrdU cell proliferation assay of corneal endothelial cells treated with deferoxamine [50 µM, 100 µM and 200 µM] or DMSO only as a control group for 24 h. Co: control; DFO: deferoxamine. *n* = nine independent experiments with four replicates per experiment.

Figure 3 indicates the proliferation of corneal endothelial cells after 24 h of incubation with deferoxamine or DMSO only (Figure 3). Whether for the treatment with 200 µM deferoxamine (98 ± 30%; *p* = 0.6950) or for 100 µM (96 ± 22%; *p* = 0.8000) and 50 µM (97 ± 21%; *p* = 0.9254), significant changes in cell proliferation could be observed when compared to the control group treated with DMSO only (100 ± 15%, Figure 3). In addition, no significant differences between the deferoxamine-treated groups were present.

### 3.4. Deferoxamine Accelerates Corneal Endothelial Wound Closure In Vitro

**Figure 4:**
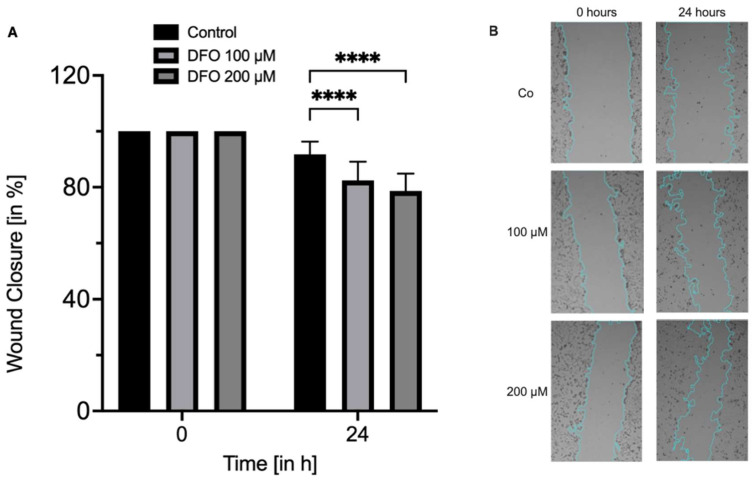
Deferoxamine accelerates corneal endothelial wound closure in vitro. (**A**) Wound closure assay of corneal endothelial cells at start (0 h) and after 24 h either treated with DMSO only or deferoxamine [100 µM and 200 µM]. (**B**) Representative images of all three groups at start and after 24 h of incubation. Displayed wound area within the blue lines. **** *p* < 0.0001. Co: control; DFO: deferoxamine. *n* = three independent experiments with five replicates per experiment.

When compared to the initial wound area, an incubation with DMSO only (control) for 24 h led to a mean size of the remaining wound area of 91.8 ± 4.5% (Figure 4A). Both 100 µM and 200 µM deferoxamine showed a significantly smaller remaining wound area of 82.4 ± 6.7% (*p* < 0.0001) and 78.7 ± 6.2% (*p* < 0.0001) in comparison to the control group after 24 h of treatment (Figure 4A). Interestingly, when treated with 200 µM of deferoxamine, the remaining wound area was significantly smaller than the one treated with 100 µM deferoxamine (*p* = 0.0446). Figure 4B depicts representative images of all three groups at the start and after 24 h of incubation (Figure 4B).

### 3.5. Deferoxamine Induces VEGF Expression in Corneal Endothelial Cells

**Figure 5:**
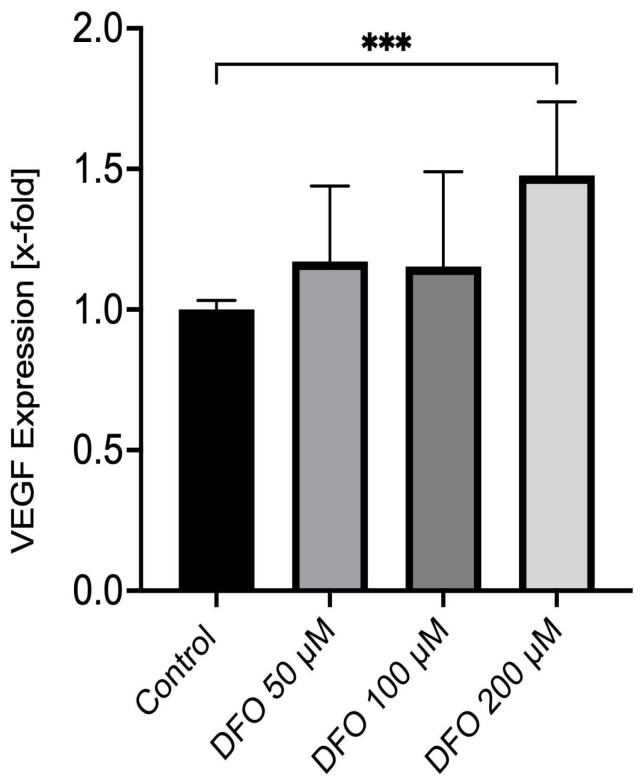
Deferoxamine induces VEGF expression in corneal endothelial cells. Real-time PCR was performed on corneal endothelial cells following incubation with deferoxamine [50 µM, 100 µM and 200 µM] or DMSO only for 12 h. *** *p* < 0.001. Co: control; DFO: deferoxamine. *n* = five independent experiments with two replicates per experiment.

In response to an incubation of corneal endothelial cells with 200 µM deferoxamine for 12 h, a significant induction of VEGF mRNA expression to 1.48- ± 0.57-fold from 1.00- ± 0.03-fold in the control group could be observed (*p* = 0.0006; Figure 5). When compared to the treatment groups with 50 µM (1.17- ± 0.16-fold) and 100 µM (1.15- ± 0.34-fold) deferoxamine, this induction was also significant (*p* = 0.0023 and *p* = 0.0098; Figure 5). No significant induction of VEGF mRNA expression could be observed between the control group and 50 µM as well as 100 µM deferoxamine (*p* = 0.9634 and *p* = 0.7492; Figure 5).

## 4. Discussion

Based on the following findings, we conclude that deferoxamine modulates corneal endothelial cell biological properties associated with an increased VEGF expression: (1) deferoxamine significantly increased cell viability while no proliferative effects could be observed; (2) deferoxamine significantly induced corneal endothelial wound closure in vitro; (3) deferoxamine significantly induced VEGF mRNA expression. Endothelial origin of the cells was suggested via immunohistochemistry for Na^+^/K^+^-ATPase. However, direct activation of HIF signaling was not assessed in this study.

As we could not observe impairing effects on proliferation or a decreased cell viability of corneal endothelial cells under the treatment of deferoxamine, we propose it as a safe substance to be administered topically in the tested concentrations. To the best of our knowledge, this has not been addressed before. In a British case series published in 1970, 36 patients were treated with deferoxamine eye drops due to residual rust following foreign body injuries of the cornea. While no acute reactions were present, two patients developed self-limiting corneal ulceration with no residual visual disturbances. However, it remains unclear whether this complication is primarily following the foreign body injury or directly linked to the administration of deferoxamine eye drops [31]. Interestingly, as described below, deferoxamine decreases cold-induced injury in porcine corneal endothelial cells [32]. Furthermore, based on those results, the working group of Radovits et al. established an improved donor medium for the preservation of kidney and liver before transplantation. By adding deferoxamine, they found a superior protection of endothelial cells from iron-dependent oxidative injury [33]. Those studies further corroborate our hypothesis on the safety of deferoxamine.

While no induction of corneal endothelial cell proliferation was observed, deferoxamine enhanced cell wound closure in higher concentrations, which can be important for corneal endothelial restoration after injury. However, the wound closure assay used in this study does not allow discrimination between increased cell migration, altered metabolic activity, or changes in cell survival. Given the observed increase in WST-1 signal, contributions from metabolic activity cannot be excluded. The lack of effect on corneal endothelial cell proliferation does not come as a surprise. Several species-specific CEC characteristics have been addressed previously. In brief, traumatic injuries, aging, inflammation, or other corneal diseases induce wound healing processes of the corneal endothelium that, in primates and felines, primarily occur via cell migration and enlargement of adjacent healthy corneal endothelial cells, rather than via cell proliferation [9,34,35,36]. In addition, analogous to our finding, three recent studies propose an induction of cell migration in a breast cancer cell line [37], in a colon cancer cell line [38] and in dental pulp cells [39] via HIF-1α in the presence of deferoxamine concentrations between 10 µM and 300 µM.

VEGF is a downstream mediator of the hypoxia-inducible factor signaling, which is upregulated under hypoxic conditions [40]. In the cornea, hypoxic stress and the presence of reactive oxygen species attenuates corneal epithelial cell growth and therefore delays corneal epithelial wound healing [41]. In addition, hypoxic conditions of the corneal epithelium during contact lens wear lead to a significant upregulation of HIF-1α and VEGF to mediate a neovascular response [42]. Corneal endothelial hypoxia affects the pump function of corneal endothelial cells, causing corneal edema due to the lack of ATP and a subsequent dyshomeostasis of ion transportation via Na^+^/K^+^-ATPase [43]. Deferoxamine significantly induced VEGF mRNA expression in corneal endothelial cells in our study. Interestingly, deferoxamine exerts neuroprotective and antiapoptotic effects and thus ameliorates traumatic brain injury in rats through HIF-1α and its downstream target gene VEGF [44]. In porcine corneal endothelial cells, deferoxamine was able to prevent the iron-dependent formation of reactive oxygen species implicated in cold-induced injury, resulting in an inhibition of the latter [32]. Because the formation of ROS is also induced by hypoxia, deferoxamine may maintain corneal endothelial integrity and water homeostasis under hypoxic conditions. Since we also found an overexpression of VEGF, further studies need to evaluate whether this effect is mediated via hypoxia-induced signaling pathways.

In vivo, corneal endothelial cells remain in a non-proliferative state as they are arrested in the G1 phase of the cell cycle [45]. Even though we suggested an endothelial origin of the cells via immunohistochemical staining for Na^+^/K^+^-ATPase and their characteristic hexagonal morphology in phase contrast microscopy, one must bear in mind that CECs can undergo endothelial-to-mesenchymal transition in vitro [46]. This process may occur in CECs in cell culture over time and eventually lead to a change in morphology and molecular biological response [47]. Nevertheless, Peh et al. found that by seeding endothelial cells in a density of over 10.000 cells/mm^2^, their morphological integrity can be maintained at least until the third passage [48]. Since we only used corneal endothelial cells in lower passage counts and seeded them densely, we do not think that endothelial-to-mesenchymal transition plays an important role in our particular study.

However, a key limitation of this study is that activation of HIF-1α signaling was not directly assessed. While VEGF is a known downstream target of HIF-1α, its expression alone is not sufficient to confirm pathway activation. Future studies should include direct assessment of HIF-1α protein stabilization, nuclear localization, or transcriptional activity. Additionally, the translational relevance of these findings is limited by the in vitro design and the use of porcine cells. Further functional endpoints such as barrier integrity, pump function, and long-term phenotype stability were not assessed.

## 5. Conclusions

In summary, our study indicates that deferoxamine enhanced the viability and wound healing of corneal endothelial cells in vitro as well as upregulating VEGF mRNA expression. However, the underlying molecular mechanism remains to be elucidated. Speculatively, hypoxia-induced signaling pathways may be involved.

## Data Availability

The data presented in this study are available on request from the corresponding author.
